# Integrated school based nutrition programme improved the knowledge of mother and schoolchildren

**DOI:** 10.1111/mcn.12794

**Published:** 2019-05-31

**Authors:** Imelda Angeles‐Agdeppa, Emilita Monville‐Oro, Julian F. Gonsalves, Mario V. Capanzana

**Affiliations:** ^1^ Department of Science and Technology Food and Nutrition Research Institute Taguig City Philippines; ^2^ International Institute of Rural Reconstruction Cavite Philippines

**Keywords:** integrated school‐based nutrition, mother's knowledge, nutrition education, nutritional status of children, school garden, supplementary feeding

## Abstract

This study evaluates the effects of nutrition education on improving knowledge, attitude, and practice (KAP) of mothers and the improvement of the nutritional status of their children. A cluster randomized controlled design using multistage sampling was employed. The integrated school‐based nutrition programme included gardening, nutrition education for parents, and supplementary feeding for children (GarNESup). KAP of mothers was assessed using pretested questionnaires administered by teachers. The randomly selected schools were randomly allocated into two groups: Both schools provided lunch to targeted children with one‐dish indigenous vegetable recipe, but School 1 received iron‐fortified rice whereas School 2 was provided ordinary rice. Eighty wasted and/or anaemic children in each school were fed for 120 days. Nutrition education for children's parents was done every school card claim day and during parent–teacher meetings using 10 developed modules. Weight, height, and haemoglobin level of children and KAP of mothers were measured at baseline and endpoint using standard techniques. KAP of mothers who had completed more than six modules had significantly increased from baseline to endpoint: Negative consequence of worm infestation (33.3% to 60.6%, *P* = 0.035), importance of serving breakfast for children (42.4% to 78.8%, *P* = 0.004), cooking vegetables (63.6% to 93.9%, *P* = 0.002), and purchasing fortified foods was recorded (51.5% to 93.9%, *P* = 0.000). Children in School 1 had significantly higher weight gain (1.33 ± 0.72) and haemoglobin level (0.49 ± 0.99) than children in School 2 (0.84 ± 0.59; 0.12 ± 0.70). Nutrition education resulted to significant increase of mother's KAP and the implementation of the integrated school‐based nutrition model significantly improved children's nutritional status.

**Key messages**

- Improved KAP of mothers as a result of nutrition education sessions.
- Linking school garden with supplementary feeding through nutrition education of parents is an effective nutrition sensitive and nutrition specific interventions to improve nutritional status of schoolchildren.
- Improved nutritional status of children as a result of the integrated school‐based nutrition programme comprising of gardening, nutrition education, and supplementary feeding.

## 1 INTRODUCTION

Nutrition‐related knowledge, attitudes, and practices (KAP) of both parents are important determinants of nutritional status and are probable contributors to malnutrition (Fathea, Salama, & Dalia, 2014). In delivering health and nutrition services, schools are appropriate platforms to reach many because parents spend time in school for parent–teacher meetings and receiving of children's school card. In the Philippines, usually, the mothers are responsible for meal preparation at home. The lack of correct KAP about nutrition among mothers may put the nutritional status of family members especially the children at risk.

In 2011, about 32% of Filipino children 5–10 years were underweight, stunted (33.6%), and wasted (8.5%) (DOST‐FNRI, 2011). Anaemia prevalence among school children aged 6–12 years is 20% (DOST‐FNRI, 2008). The Department of Education (DepEd) instituted a school nutrition programme that included a regular school‐based feeding programme and a school garden programme called *Gulayan sa Paaralan Program*. The school‐based feeding program was first launched in 1997 to address short‐term hunger among public schoolchildren. In 2011, it was revitalized targeting the severely wasted children from kindergarten to Grade 6 with funding support from the national government. The 120‐day revitalized feeding programme is expected to improve the nutritional status from severely wasted to normal status, improve classroom attendance by 85% to 100%, and improve the children's health and nutrition values and behaviour (DepEd Order No. 37 S; Department of Education, 2012). The 2013 Global Child Nutrition Forum meeting in Salvador, Brazil, recommended the “School Feed Program” be considered a key national investment that complements early child interventions to promote full child development. It was also recommended that school feeding programmes be integrated with effective complementary interventions including nutrition education.

A common nutrition intervention that aims to address gaps in KAP is nutrition education. Nutrition education is a set of learning experiences designed to facilitate voluntary adoption of eating and other nutrition‐related behaviour conducive to health and well‐being that includes those on a limited budget (Contento, Manning, & Shannon, 1992). In the Philippines, this type of nutrition intervention has been widely used due to the high‐rate efficiency, which led to the implementation of various nutritional education programmes, one of which is the Nutrition and Health Kiddie Class that aims to educate children (4–6 years) about the importance of food and nutrition (NFP, 2015). Additionally, in a study by Angeles‐Agdeppa, Saises, and Capanzana (2012) that modelled the scaling‐up of the rice fortification programme in the Philippines, significant increases in the percentages on parent's knowledge about iron were observed from baseline to endpoint: knowing what is iron (30.2% to 97.5%, *P* ≤ 0.001), identifying good food sources of iron (28.3% to 95.0%, *P* ≤ 0.001), and what iron‐fortified rice (IFR) is and can do (5.6% to 81.9%, *P* ≤ 0.001). A significant increase in the practice of buying IFR was also observed from baseline to endpoint (2.4% to 51.4%, *P* = 0.041).

Internationally, a programme by Cornell University, Ithaca, New York, on International Nutrition uses nutrition education among children or parents as one of their tools in mitigating malnutrition and hunger (Cornell University, 2014). Improved nutrition education has been one of the key factors to prevent 12,000 deaths a year worldwide according to the UN Millennium Campaign (United Nations, 2012).

This study evaluates the effects of nutrition education on improving KAP of mothers and the improvement of the nutritional status of children as a result of the implementation of the school‐based nutrition programme. The results of this study could serve as a model for administering nutrition education for mothers in the schools as an integral component for scaling up the school‐based nutrition programme that effectively links school garden produce to school feeding (GarNeSup).

## 2 METHODS

### 2.1 Study design and study sites

A cluster randomized controlled design using multistage sampling was employed in this study. It was conducted in the province of Cavite with a total population of 2,856,765 and an area of 1,297.6 km^2^. DepEd Cavite province had 27 school districts with 280 public elementary schools. All schools were clustered per district. In each district, schools were classified as with and without school gardens. About 72 (40%) have school gardens, but only 27 schools or one per district were practicing biointensive gardening (BIG). Schools were then stratified based on population either <700 or >700 schoolchildren. The study sites were two schools randomly selected from the 15 schools with >700 schoolchildren.

### 2.2 Sample size

The sample size was calculated based on mean weight of 18.0 and 1.7 kg *SD* as reference value. Assuming a minimum increase of 1.2 kg *SD*, 90% power test at 5% level of significance, the sample would be 42 children for each group (Angeles‐Agdeppa, 2006). However, because the study has other variables to measure (e.g., haemoglobin) where variability could be high, and to allow higher attrition rate, the sample size per group was increased to 80 children in each school.

The inclusion criteria set for this study were schoolchildren aged 6 to 8 years old, underweight with weight‐for‐age *z*‐score less than 2 *SD* (WAZ < −2 *SD*) and/or anaemic as indicated by haemoglobin level < 12 g/dl (WHO, 2001), with parental consent, and not a participant of other feeding programmes. A total of 908 schoolchildren (School 1, *N* = 420; School 2, *N* = 488,) were screened for underweight and anaemia. Eighty (80) children in each school were selected on an enrolment basis and served as participants of this study.

### 2.3 Supplementary feeding

A 3‐week cycle menu was developed by the Department of Science and Technology–Food and Nutrition Research Institute (DOST‐FNRI) specifically for this study. These menus consisted of standardized recipes of indigenous vegetables from the school gardens. The nutrient contents of the recipes were computed to meet at least 30% of the daily nutrient requirement specifically for iron and vitamin A. However, because taste and palatability was one of the criteria for recipe development, some recipes fall short of achieving 30% of the daily requirement for these two micronutrients. These recipes had undergone acceptability and liking assessment by trained taste panellist of DOST‐FNRI and by some schoolchildren. Modifications were done based on panellists' comments and children's leftover (Angeles‐Agdeppa et al., 2018).

Schools 1 and 2 have provided free lunch with a standardized one‐dish indigenous vegetable recipe, but School 1 received IFR and School 2 got the ordinary rice. A 3‐week cycle menu that included recipes of indigenous vegetables that were available in school gardens were developed as guide for the daily feeding. A cup of IFR provided an additional 0.6 mg of iron in School 1 compared with ordinary rice in School 2 (Angeles‐Agdeppa et al., 2018).

Food purchasing and meal preparation were done by a teacher assigned as feeding coordinator assisted by volunteer parents. Children in each school were fed lunch daily for 120 days during school day in a designated feeding room under the supervision of trained research assistants.

Baseline and endpoint measurements were conducted for weight, height, and haemoglobin level. Weight of children was measured using a Detecto weighing scale (Webb City, Mo. U.S.A.) whereas height was measured using a microtoise (Depose, France) posted flat against the wall. All children were in light clothing and barefooted during measurements. Measurements were taken twice by trained research assistants to validate the accuracy then take the average. All equipment was calibrated every measurement day. Underweight was defined as weight‐for‐age *z*‐score less than 2 *SD* (WAZ < −2 *SD*), stunting was height‐for‐age *z*‐score less than 2 *SD* (HAZ < −2 *SD*), and wasting was weight‐for‐height *z*‐score less than 2 *SD* (WHZ < −2 *SD*; WHO, 2007).

Nonfasting whole blood (about 5 ml) was collected via venipuncture by experienced registered medical technologists from the DOST‐FNRI. All samples were collected in the morning at the schools and were processed immediately within 4 hr after blood extraction using a portable spectrophotometer by cyanmethemoglobin method. In this study, haemoglobin cut‐off point for anaemia at sea level was used; hence, a haemoglobin concentration of >70 to <120 g/L was considered anaemic (WHO, 2001).

### 2.4 Nutrition education

Mothers of the schoolchildren in School 1 and School 2 were the participants of nutrition education. The research team developed 10 modules on health and hygiene, nutrition, and gardening for use during nutrition education sessions. Trained teachers administered the lectures during parent–teacher meetings usually done when there was a need to solicit parent's participation in school activities. Another avenue for educating parents was during card day when parents go to school to personally receive their child's school performance card. In the Philippines, mothers do this responsibility. Moreover, mothers were also tasked to do meal planning, preparation, and cooking. In an effort to observe gender sensitivity in this study, the letter of invite to attend the nutrition education classes was addressed to both parents. However, only 145 mothers have responded. Mothers were encouraged to visit the school garden and read various types of information, education, and communication materials such as flyers, theme posters, and billboards posted in the school premises. These were developed by the project team specifically for the study. Baseline and endpoint KAP were assessed using pretested questionnaires, which consisted of 15 items each for knowledge, attitude, and practice. Every item was a closed‐ended statement asking the respondent to answer “Tama” if they thought the statement was correct, “Mali” if they thought the statement was wrong, and “Hindi Alam” if uncertain of the answer. Also, every nutrition education session, the teachers administered a pre–post‐test contained in each module. This study arbitrarily considered completing six modules as high attendance in nutrition education. Those who have attended 1 to 5 modules were considered as low attendance in nutrition education.

### 2.5 Gardening

The International Institute for Rural Reconstruction led the technical assistance in the implementation of schools garden using the BIG approach. Schools were given garden implements so that gardens will be maintained. Teachers assigned as garden coordinators were trained and oriented about the project. The BIG approach features key principles and practices that promote garden health and resilience: School gardens must use organic fertilizers (*kakawate*‐based), must have at least 200‐m^2^ garden bed and deep dug at least 1 ft, good water source, proper drainage system, and use mulch to protect soil. Daily produce from the gardens, usually leafy greens, were used in the supplementary feeding following the developed vegetable recipes. Different types of vegetables from the gardens that were used in the supplementary feeding were weighed and recorded. Total weight was the sum of the daily harvest and was costed based on current price of the vegetables in the locality at a given period.

### 2.6 Limitations

The process and manner how teachers delivered the lectures to children was not observed; hence, quality of teaching and actual time allotted were not directly observed and recorded by the research team.

### 2.7 Ethical consideration

The study was approved by the Food and Nutrition Research Institute Institutional Ethics Review Committee (FIERC Registry No. 2012‐06‐20‐0006‐2) and was carried out in accordance with the Declaration of Helsinki, guided by the Council for International Organizations of Medical Sciences Ethical Guidelines for Biomedical Research Involving Human Subjects (CIOMS, 2002) and the National Guidelines for Biomedical/Behavioral Research (PNHRS, 2011). The parents were clearly informed of the objectives, procedures, risks, and benefit that their children may encounter by participation in the study. Individual signed parental informed consent forms was obtained. Children aged ≥7 years were asked to sign an assent form.

Any adverse events (AE) and/or complaints experienced by the children based on self‐reported incidents were recorded in a prescribed AE form. As per protocol, any reported AE, such as abdominal pain, diarrhoea, or gastric irritation, must be referred to the study physician. However, during the course of the study, no serious AE were reported.

All children identified as severely anaemic (Hb <7 g/dl) during screening and those children who remained anaemic after the study were referred to the nearest government health facility for further management. Children in School 2 were given 5 kg of IFR at end of the study.

### 2.8 Statistical analysis

Data encoding and checking was done using Epi‐Info Version 3.5.1. Descriptive statistics such as frequencies, percentage, mean, and standard deviation were also derived using the same software.

Shapiro–Wilk test was done to test the normality of the data (e.g., anthropometric measures, and knowledge scores). Independent *t*‐test was performed to determine the significant differences between schools (e.g., child anthropometric measures and scores). Paired *t*‐test was used to determine the significant difference within groups. Fisher exact test was performed to determine the significant difference of the proportion between schools and McNemar change test was done to determine the significant change in the proportion between time periods. Spearman correlation was calculated to test the association of the two variables (e.g., nutritional status of child and knowledge of the mother).

## 3 RESULTS

### 3.1 Cost/saving from the school garden

There was significant increase in the types of vegetables used in the feeding programme from baseline to endpoint (from 7 to 10, *P* = 0.000). The total amount of vegetables harvested from the school gardens for use in the supplementary feeding ranged from 68 to 110.28 kg for the whole 120 days with a total cost of PhP 3,282.90 to 5,568.63.

### 3.2 Socio‐economic demographics and other information

Most of the participants belonged to a single/nuclear family (61.9%) with a mean household size of 6. The mean monthly family income of School 1 and School 2 was PhP 9,896.92 ± 5,870.64 and PhP 12,023.98 ± 11,800.86, respectively. And the mean daily food expenditure was PhP 225.65 ± 103.19. About 44% to 48% of the households earned below PhP 1,577.92 per capita per month, which is classified as poor (NSCB, 2012).

Most of the father (60.0%) and mother (60.6%) had high school education, and the major occupation was either skilled related work (32.5%) or unskilled related work (30.5%).

The age of children was similar between schools with a mean of 6.71 ± 0.78 years. Distribution of children by grade was not significantly different between schools (*P* = 0.207). Most of the children who participated in the study were Grade 1 (41.9%).

The proportion of children who were dewormed in School 1 and School 2 was 76.2% and 83.8%, respectively. The proportion distribution of children who were dewormed was not significantly different between schools (*P* = 0.236).

Of the total 160 children qualified to participate in the study, a complete data set from baseline to endpoint was only obtained from 146 children. Reasons for dropouts were transferred residence (School 1 = 8.8%; School 2 = 7.5%) or sickness/illness (School 2 = 1.2%).

### 3.3 Nutritional status

At baseline, the mean weight and height values were similar in both schools. Mean weight and height of children had significant increase in School 1 and School 2 from baseline to endpoint; however, the mean increase in weight in School 1 was significantly higher (1.33 ± 0.72, *P* = 0.0134) than in School 2 (0.84 ± 0.59) (Table 1). Moreover, significant decreases were observed in the prevalence of underweight children in School 2 (56.2% to 34.2%, *P* = 0.002) and prevalence of stunting in School 1 (43.8% to 26%, *P* = 0.004) from baseline to endpoint (Table 2).

**Table 1:** Anthropometric measurements and haemoglobin level of children at baseline and endline by school

Measurement	School 1 Mean (±*SD*)	School 2 Mean (±*SD*)	Difference	*P* value
Weight (kg)				
Baseline	16.56 (±1.71)	16.90 (±1.69)	0.34	0.255
Endline	17.90 (±1.86)	17.74 (±1.73)	0.16	0.592
Difference	1.33 (±0.72)	0.84 (±0.59)	0.49	0.013*
*P* value	0.000*	0.000*		
Height (cm)				
Baseline	110.32 (±5.37)	109.92 (±5.13)	0.40	0.637
Endline	113.67 (±5.50)	113.08 (±5.24)	0.59	0.513
Difference	3.35 (±0.60)	3.16 (±0.63)	0.19	0.728
*P* value	0.000*	0.000*		
Haemoglobin (g/dl)				
Baseline	12.60 (±0.96)	12.52 (±0.77)	0.08	0.602
Endline	13.09 (±0.71)	12.64 (±0.86)	0.45	0.001*
Difference	0.49 (±0.99)	0.12 (±0.70)	0.37	0.012*
*P* value	0.000*	0.140		

* Significant at 0.05.

**Table 2:** Prevalence of underweight, stunting, wasting, and anaemia among children at baseline and endpoint by school

Nutritional status	School 1 (*n* = 73)	School 2 (*n* = 73)	*P* value
	*n* (%)	*n* (%)	
Underweight			
Baseline	26 (35.6)	41 (56.2)	0.013*
Endline	24 (32.9)	25 (34.2)	0.861
*P* value	0.824	0.002*	
Stunted			
Baseline	32 (43.8)	27 (37.0)	0.399
Endline	19 (26.0)	18 (24.7)	0.849
*P* value	0.004*	0.078	
Wasted			
Baseline	6 (8.2)	11 (15.1)	0.197
Endline	8 (11.0)	11 (15.1)	0.461
*P* value	0.727	1.000	
Anaemia			
Baseline	48 (65.8)	47 (64.4)	0.862
Endline	35 (47.9)	45 (61.6)	0.862
*P* value	0.000*	0.500	

* Significant at 0.05.

At baseline, mean haemoglobin level of children was similar in both schools. Significant increase in the mean Hb levels in School 1 (0.49 ± 0.99, *P* = 0.000) at the end of supplementary feeding. The increase in the mean Hb level in School 1 (0.49 ± 0.99) was significantly higher (*P* = 0.032) than School 2 (0.12 ± 0.79). Anaemia prevalence in School 1 (65.8% to 47.9%, *P* = 0.000) significantly decreased from baseline to endpoint (Table 2).

### 3.4 Nutrition education

Of the 160 mothers of children invited for the nutrition education activity, 145 mothers gave consent to participate, hence, have baseline data. However, only 105 mothers had participated and completed at least six modules, and 45 mothers had attended one to four modules.

From baseline to endpoint, among mothers who had high attendance in nutrition education, there was a significant percentage increase in knowledge of mothers on the importance of hygiene and grooming specifically on knowing the negative consequence of worm infestation (33.3% to 60.6%, *P* = 0.035) and the importance of meal preparation specific to knowledge on serving breakfast for children (42.4% to 78.8%, *P* = 0.004).

The attitude on meal preparation had significantly improved from baseline to endpoint as indicated by increases in the percentage of mothers preparing nutritious foods (18.2% to 42.4%, *P* = 0.039) and cooking vegetables (63.6% to 93.9%, *P* = 0.002).

Improved practices from baseline to endpoint were observed: It was easy for the mothers to convince their children to eat vegetables (36.4% to 69.7%, *P* = 0.003); they do not need to force their children to eat vegetables (9.1% to 30.3%, *P* = 0.039). Also, a significant improvement in the practice of purchasing fortified foods was recorded (51.5% to 93.9%, *P* = 0.000). On meal preparation, they practice washing the vegetables before use (27.3% to 87.9%; *P* = 0.000). Among those who had low attendance in nutrition education, no significant improvement was observed in their KAP (Table 3).

**Table 3:** Percentage of parents with correct knowledge, attitude, and practice about nutrition from baseline to endpoint

	LANE (*n* = 40)			HANE (*n* = 105)		
	Baseline	Endline	*P* value	Baseline	Endline	*P* value
Knowledge						
Basic nutrition						
Nutrient content of green and leafy vegetables	92.3	94.9	1.000	93.9	93.9	1.000
Consumption fruits and vegetables three times a week	20.5	23.1	1.000	18.2	21.2	1.000
Fruits and vegetables can improve health and prevent sickness	97.4	97.4	1.000	93.9	100.0	0.500
Hygiene and grooming						
Personal hygiene	97.4	100.0	1.000	97.0	100.0	1.000
Having worms	41.0	35.9	0.774	33.3	60.6	0.035*
Backyard gardening						
Planting vegetables	53.8	59.0	0.727	78.8	93.9	0.125
Use of chemical fertilizer	51.3	61.5	0.424	39.4	24.2	0.267
Food fortification						
Iron deficiency	51.3	66.7	0.210	72.7	84.8	0.289
Vitamin A deficiency	74.4	82.0	0.508	75.8	93.9	0.109
Food fortification	61.5	69.2	0.648	66.7	54.5	0.424
Good nutrition						
Nutrition	7.7	7.7	1.000	18.2	18.2	1.000
Enough food intake	74.4	71.8	1.000	81.8	84.8	1.000
Lack of nutrition	92.3	94.9	1.000	90.9	100.0	0.250
Food groups	97.4	97.4	1.000	97.0	90.9	0.500
Meal preparation						
Serving breakfast	41.0	35.9	0.774	42.4	78.8	0.004*
Attitude						
Basic nutrition						
Protein content of dried beans	28.2	30.8	1.000	33.3	30.3	1.000
Habit in eating a variety of foods	20.5	28.2	0.508	21.2	39.4	0.070
Food diversity	74.4	69.2	0.754	63.6	63.6	1.000
Hygiene and grooming						
Sanitation in cooking	100.0	97.4	1.000	100.0	100.0	1.000
Backyard gardening						
Home gardening in providing fresh vegetables	97.4	97.4	1.000	97.0	100.0	1.000
Food fortification						
Food products with “Sangkap Pinoy Seal”	35.9	41.0	0.815	51.5	66.7	0.332
Good nutrition						
Eating fruits and vegetables	100.0	100.0	1.000	97.0	100.0	1.000
Feeding programme	100.0	97.4	1.000	100.0	100.0	1.000
Eating food rich in protein	64.1	53.8	0.388	69.7	69.7	1.000
Eating healthy snacks	89.7	87.2	1.000	87.9	87.9	1.000
Importance of food diversity	100.0	97.4	1.000	100.0	100.0	1.000
Being a good model in eating fruits and vegetables	100.0	97.4	1.000	100.0	100.0	1.000
Meal preparation						
Preparing nutritious food	7.7	20.5	0.180	18.2	42.4	0.039*
Right way of preparing vegetables for cooking	66.7	64.1	1.000	63.6	93.9	0.002*
Right way of cooking vegetables	100.0	94.9	0.500	97.0	100.0	1.000
Practices						
Nutrient content of food						
Convincing their children in eating vegetables	41.0	48.7	0.581	36.4	69.7	0.003*
Buying fruits and vegetables	100.0	76.9	0.004*	100.0	90.9	0.250
Forcing their children in eating vegetables	12.8	10.2	1.000	9.1	30.3	0.039*
Hygiene and grooming						
Letting their children having a long nail	94.9	89.7	0.625	100.0	93.9	0.500
Backyard gardening						
Having a backyard garden	59.0	61.5	1.000	48.5	51.5	1.000
Food fortification						
Looking food products with “Sangkap Pinoy Seal”	48.7	53.8	0.804	51.5	93.9	0.000*
Good nutrition						
Decision making in serving food	41.0	51.3	0.455	54.5	57.6	1.000
Meal preparation						
Preparing nutritious foods for snacks	66.7	69.2	1.000	66.7	81.8	0.180
Buying cooked food	89.7	74.4	0.070	90.9	97.0	0.500
Cutting vegetable first before washing it	30.8	41.0	0.344	27.3	87.9	0.000*
Giving money to children for their snacks	35.9	46.2	0.344	51.5	45.4	0.625
Putting a little oil in cooking vegetables	87.2	76.9	0.344	75.8	87.9	0.344
Cooking the vegetables very well	12.8	28.2	0.180	15.2	33.3	0.146
Serving vegetables at home	69.2	76.9	0.549	63.6	75.8	0.388

*Note*. HANE: high attendance in nutrition education; LANE: low attendance in nutrition education.

* Significant at 0.05.

Table 4 shows a significant increase in the mean scores of pre–post‐test of parents concerning lessons on constraints and challenges in understanding nutrition (3.0 to 3.5, *P* = 0.000), sustaining nutrition in home settings (0.5 to 4.4, *P* = 0.000), proper nutrition guidelines and nutrition practices (4.4 to 6.0, *P* = 0.002), complimentary feeding (1.6 to 5.6, *P* = 0.000), encouraging children to eat vegetables (3.9 to 5.7, *P* = 0.000), vegetable preparation and cooking (2.6 to 6.0, *P* = 0.000), food fortification (3.3 to 6.1, *P* = 0.000), guide for vegetable garden (2.6 to 6.1, *P* = 0.000), and personal hygiene and health (4.4 to 5.9, *P* = 0.000).

**Table 4:** Comparisons of mean scores of preexamination and postexamination of mothers by module

Module	Base	End	*P* value
Understanding nutrition	3.0	3.5	0.000*
Food needed by school children	5.6	5.6	0.425
Constraints and challenges in sustaining nutrition in home setting	0.5	4.4	0.000*
Proper nutrition guidelines and nutrition practices	4.4	6.0	0.000*
Complementary feeding	1.6	5.6	0.000*
Encouraging children to eat vegetables	3.9	5.7	0.000*
Vegetable preparation and cooking	2.6	6.0	0.000*
Food fortification	3.3	6.1	0.000*
A guide for vegetable gardens	2.6	6.1	0.000*
Personal hygiene and health	4.4	5.9	0.000*

* Significant at 0.05.

The knowledge and practices of the mothers showed significant relationship to the weight of the children (*P* < 0.05) (Table 5).

**Table 5:** Association between nutritional status and postscores of mothers in knowledge, attitude, and practices in nutrition

		Weight status	Height status	BMI status
Knowledge	Coefficient	0.184	0.057	0.119
	*P* value	0.007*	0.404	0.084
Attitude	Coefficient	0.066	0.021	0.012
	*P* value	0.332	0.750	0.860
Practices	Coefficient	0.136	−0.040	0.003
	*P* value	0.046*	0.564	0.967

*Note*. BMI: body mass index.

* Significant at 0.05.

## 4 DISCUSSION

Parents are mostly responsible on their children's eating behaviours and preferences. Parents create environments for children that may foster the development of healthy eating behaviours and weight or that may promote overweight and aspects of disordered eating (Scaglioni, Salvioni, & Galimberti, 2008). Especially, mothers are the role models of their children about eating behaviours (Nurcan, Kisac, & Karakuş, 2014). It is assumed that nutritional knowledge level of the mother could be effective on eating behaviours of their children. The school is an opportune setting to provide health and nutrition services to parents.

This study evaluates the effects of nutrition education on improving KAP of mothers and the improvement of the nutritional status of their children who participated in the integrated school‐based nutrition programme. The integrated school‐based nutrition programme consists of gardening, nutrition education, and supplementary feeding (GarNeSup). The purpose of the garden in the school programme or locally termed as *Gulayan sa Paaralan Program* of the DepEd is to supplement the vegetables needed in the supplementary feeding programme; however, this is seldom if not being practised at all. This disconnect between these two programmes might be attributed to reasons such as the unacceptability of schoolchildren to eat indigenous vegetables that are usually grown in school gardens. These vegetables are grown because of their resilience to pest infestation and are considered climate smart vegetables because they do not need a lot of resources in order to grow. Another reason is that gardens are not maintained; hence, no or limited produce are obtained. With this study, garden produce have supplemented the vegetables needed in the feeding programme. For children to like vegetables, recipes were developed to be more appealing and palatable to children. Linking these two programmes could have direct economic benefits and can potentially benefit the sustainability of the programmes (WFP, 2013). On the other hand, nutrition education among parents is not a regular programme of the DepEd. Although the school is providing nutritional interventions to children, the mother's nutrition knowledge applied in daily life at home greatly affects the condition of the family nutrition (Dadang & Faisal, 2015) should be a cause of concern. It is in this context that the project developed nutrition modules for use during learning sessions.

Taking into account the benefits of gardening and supplementary feeding as a components of the GarNeSup project showed significant increments in mean weight and height in both schools which translated to significantly reduced prevalence of underweight in School 2 whereas significant decline in stunting prevalence (43.8% to 26%, *P* = 0.004) in School 1. The add‐on benefit of consuming IFR in School 1 revealed a significant increase in Hb level (0.49 ± .99; *P* = 0.000) and consequently reduced anaemia prevalence (65.8% to 47.9%, *P* = 0.000). The reduction of anaemia prevalence in School 1 could be attributed to an increased iron intake from the IFR apart from the iron provided by the enhanced indigenous vegetable recipes from the school gardens. The use of IFR in battling Iron Deficiency Anaemia (IDA) has also proven in numerous studies in the Philippines (Angeles‐Agdeppa, Capanzana, Barba, Florentino, & Takanashi, 2008; Angeles‐Agdeppa, Saises, Capanzana, Juneja, & Sakaguchi, 2011; Angeles‐Agdeppa, Magsadia, & Capanzana, 2015).

The results of the present study is in congruent with the findings of a systematic review that showed that providing supplementary food to young children in low‐ and middle‐income countries had small but statistically significant positive effects on weight and height (0.12‐kg gain for weight and 0.32‐cm increase in height over 6 months in the most rigorous trials). Positive effects were also seen for other physical outcomes such as height‐for‐age scores, weight‐for‐height scores, and haemoglobin levels (Kristjansson et al., 2007). However, weight gain in this present study revealed higher increments in both School 1 and School 2 (1.33 and 0.84 kg) and higher height gain with 3.35 cm in School 1 and 3.16 cm in School 2. The differences in findings might be due to difference in age of beneficiaries wherein the age group in the systematic review was 3‐ to 5‐year olds whereas 6‐ to 8‐year olds in the present study. Another factor would be the energy and nutrient content of the meal. In this study, the meal provided at least 272 kcal, an average iron content of 4.3 mg and vitamin A content of 453 μg Retinol Equivalent (RE). Evidence from around the world on locally sourced school meals reveals a multiple‐win opportunity for policymakers with important benefits for school achievement, employment, and national economic growth. Providing nutritionally balanced school meals with complementary nutrition education and health measures can deliver improved school performance, nutrition literacy, and employment and income in later life (Global Panel, 2015).

Poor maternal education (formal and informal) has been identified as a major constraint to good childcare practices in Ghana (Johnson et al., 1994). A well‐resourced, targeted, and coordinated nutrition education can improve maternal nutritional knowledge, health care‐seeking behaviours, and practices significantly. Effective utilization of knowledge and skills gained from health and nutrition education is, therefore, expected to improve the health and nutritional status of children through improved knowledge and care practices (United Nations, 1992). The nutrition education conducted in this study revealed an improvement in knowledge of mothers; however, some were not translated to positive attitude and practice. In almost all the nutrition modules, the posttests results of the mothers showed a significant result. Moreover, a significant positive relationship between the scores on nutritional knowledge and practices of mothers and the weight status of the child is noted in this study (*P* = 0.007; *P* = 0.046), respectively. The result is similar in previous published studies wherein some studies have reported that maternal nutritional knowledge is positively associated with the nutritional status of children (Appoh & Krekling, 2005; Dadang & Faisal, 2015; Saaka, 2014); others have also shown that adequate knowledge per se is not always translated into appropriate actions (Fathea et al., 2014; Waihenya, Kogi‐Makau, & Muita, 1996). Understanding the factors that determine the translation of adequate child health and nutritional knowledge into appropriate actions might help design more effective interventions against malnutrition. It remains unclear whether giving mothers adequate knowledge on proper childcare practices has an independent impact on child growth (Saaka, 2014).

## 5 CONCLUSION

Nutrition education resulted to significant increase of mother's KAP and the implementation of the integrated school‐based nutrition model significantly improved children's nutritional status. Using IFR in the feeding programme adds up to the beneficial effect in reducing the prevalence of anaemia. Programme implementers should not neglect the importance of nutrition education of parents as an integral activity of any interventions aiming at improving child's nutritional and health status because parents, specifically mothers, are responsible in creating environments for children that may foster healthy eating behaviours.

## CONFLICTS OF INTEREST

The authors declare that they have no conflicts of interest.

## CONTRIBUTIONS

IAA and EMO designed the study with inputs from JFG and MVC. Analysis of the data and writing of the manuscript by IAA with inputs from EMO, JFG, and MVC.
